# Interplay between the Ubiquitin Proteasome System and Ubiquitin-Mediated Autophagy in Plants

**DOI:** 10.3390/cells9102219

**Published:** 2020-10-01

**Authors:** Tong Su, Mingyue Yang, Pingping Wang, Yanxiu Zhao, Changle Ma

**Affiliations:** Shandong Provincial Key Laboratory of Plant Stress, College of Life Sciences, Shandong Normal University, Jinan 250014, China; sut0229@sdnu.edu.cn (T.S.); yangmingyue1@stu.sdnu.edu.cn (M.Y.); pingping.wang@sdnu.edu.cn (P.W.); zhaoyx@sdnu.edu.cn (Y.Z.)

**Keywords:** autophagy, degradation, the ubiquitin-proteasome system, ubiquitin, plants

## Abstract

All eukaryotes rely on the ubiquitin-proteasome system (UPS) and autophagy to control the abundance of key regulatory proteins and maintain a healthy intracellular environment. In the UPS, damaged or superfluous proteins are ubiquitinated and degraded in the proteasome, mediated by three types of ubiquitin enzymes: E1s (ubiquitin activating enzymes), E2s (ubiquitin conjugating enzymes), and E3s (ubiquitin protein ligases). Conversely, in autophagy, a vesicular autophagosome is formed that transfers damaged proteins and organelles to the vacuole, mediated by a series of ATGs (autophagy related genes). Despite the use of two completely different componential systems, the UPS and autophagy are closely interconnected and mutually regulated. During autophagy, ATG8 proteins, which are autophagosome markers, decorate the autophagosome membrane similarly to ubiquitination of damaged proteins. Ubiquitin is also involved in many selective autophagy processes and is thus a common factor of the UPS and autophagy. Additionally, the components of the UPS, such as the 26S proteasome, can be degraded via autophagy, and conversely, ATGs can be degraded by the UPS, indicating cross regulation between the two pathways. The UPS and autophagy cooperate and jointly regulate homeostasis of cellular components during plant development and stress response.

## 1. Introduction

Over the course of plant development from germination to senescence, plant morphology and metabolism change dramatically, resulting in accumulation of many obsolete proteins and cellular structures that need to be degraded. Moreover, as sessile organisms, plants must adapt to constantly changing environmental conditions [1].

In all eukaryotes, the ubiquitin-proteasome system (UPS) and autophagy are the two major protein quality control pathways utilized to degrade misfolded or redundant proteins to maintain the homeostasis of the plant proteome [2]. The UPS and autophagy pathways also play crucial roles in regulating a wide range of cytological and physiological processes by selectively removing regulatory proteins after they are no longer needed, such as those involved in plant development and stress response [3]. In the UPS, proteins to be degraded are labeled with ubiquitin molecules. This ubiquitin conjugation process requires three types of ubiquitin enzymes: E1 (ubiquitin activating enzyme), E2 (ubiquitin conjugating enzyme), and E3 (ubiquitin protein ligase) [4]. Subsequently, the poly-ubiquitinated proteins are degraded in the proteasomes, which are composed of a variety of subunits and regulatory proteins [5]. In eukaryotes, autophagy, mediated by a set of core autophagy-related (ATG) proteins, is a membrane trafficking system used to degrade protein aggregates and cytoplasmic components in the vacuole (yeast and plant) or lysosome (mammals) [6,7]. Recycling of nutrients through autophagy is essential for plant survival under starvation conditions [8]. Although both the UPS and autophagy pathways play important roles in plant growth, development, and stress response, they were long viewed as independent, parallel systems with no point of intersection. Recently, growing evidence has demonstrated that these two degradation pathways are interconnected. In this manuscript, we review the recent advances regarding the roles of the UPS and autophagy in stress response and their interconnectedness.

## 2. Ubiquitin Proteasome System

The UPS consists of two successive processes: (1) the attachment of a chain of ubiquitin molecules to the targeted protein and (2) degradation of the targeted protein by the 26S proteasome with recycling of the ubiquitin molecules [9]. During the first step, the enzymatic cascade consisting of E1, E2, and E3 is essential for ubiquitin attachment [3].

### 2.1. Ubiquitin-Proteasome System

Ubiquitin was discovered in the early 1980′s as a reusable recognition signal for selective protein degradation and is a highly conserved protein found in all eukaryotic organisms and tissues [10,11]. Ubiquitin is encoded by multiple genes as a precursor protein and undergoes proteolytic processing to produce mature ubiquitin consisting of 76 amino acids and a diglycine (Gly75-Gly76) sequence in the carboxyl-terminus [3].

Ubiquitination of proteins is a multistep process in all eukaryotes. First, the C-terminal carboxyl group of ubiquitin is connected to the conserved active-site cysteine (Cys) residue on the E1, forming an E1-ubiquitin thioester in an energy-dependent manner. Then, ubiquitin is transferred from E1 to an active cysteine residue on the E2, forming an E2-ubiquitin thioester-linked intermediate. Finally, the ubiquitin is transferred from E2 to a lysine (Lys) residue on the substrate protein by catalysis of E3 (monoubiquitination) [12]. Generally, monoubiquitination has been considered to be non-proteolytic and plays roles in the regulation of interactions and activation of substrates. However, there is evidence in yeast that monoubiquitination can also lead to the degradation of some substrates and monoubiquitination-dependent degradation is more widespread than previously assumed [13]. In addition, in plants, a single ubiquitin molecule can be attached to multiple Lys residues (multimonoubiqutination) or generate a polyubiquitin chain on a single Lys (polyubiquitination) on the target protein [14,15]. It has been shown that ubiquitinated Lys residues tend to reside within intrinsically disordered regions of the substrate proteins in plants [14]. There are seven conserved Lys residues on ubiquitin which are used to form an isopeptide bond with Gly76 of another ubiquitin molecule. In *Arabidopsis*, Lys48 is used the most frequently to form polyubiquitin chains [14,15]. Ubiquitin-modified proteins can be deubiquitinated by deubiquitinating enzymes (DUBs), which hydrolyze the isopeptide bond between ubiquitin and the substrate Lys residues [16]. Therefore, ubiquitination is reversible, making it a controllable process.

### 2.2. Enzymes of the UPS

In the *Arabidopsis* genome, there are two E1 encoding genes, 37 E2 encoding genes, and more than 1400 genes encoding E3 ubiquitin ligases or the components of the E3 complex [3,17,18]. E1s are encoded by *Ubiquitin Activating 1* (*UBA1*) and *UBA2*, which share similar sequences and expression patterns [17]. However, their function may not be completely redundant, as evidenced by the finding that mutation of *Arabidopsis UBA1*, but not *UBA2*, results in defects in innate immunity [19].

E2 is characterized by the UBC (ubiquitin conjugating) domain containing an active cysteine to form thioester linkages with E1 and E3 [20]. The 37 ubiquitin E2s, AtUBC1 to AtUBC37, are classified into 14 subgroups according to the amino acid sequences of the UBC domain. The largest subgroup, V1, contains 8 members, in which UBC8, UBC10, and UBC11 are the most active [18]. The *Arabidopsis* genome also encodes another 11 UBC-domain containing proteins. Some of these proteins are ubiquitin-conjugating enzyme variants (UEVs) that can form complexes with ubiquitin E2s to promote substrate ubiquitination [18,21]. For example, the UEV COP10 can enhance the activities of UBC8 and UBC9 to promote the formation of Lys48- and Lys63-linked ubiquitin chains [21].

The substrate specificity of ubiquitination is primarily determined by E3; thus, the E3 ubiquitin ligases are diverse. E3s are classified into three types based on the domain they use to interact with the E2-ubiquitin intermediate: Really Interesting New Gene (RING) proteins, Homology to E6-Associated Carboxyl-Terminus (HECT) proteins, and U-box domain proteins [9]. The *Arabidopsis* genome encodes about 490 RING proteins, making it the largest E3 subfamily [22]. The E3s work in two ways: by facilitating the direct transfer of ubiquitin from E2 to the substrate such as for the RING and U-box E3s, or by accepting ubiquitin from E2, forming a thioester-linked E3-ubiquitin intermediate, and then transferring the ubiquitin to the substrate such as for the HECT E3s [9]. The E3 ubiquitin ligases can interact with themselves or with other E3s to form homomeric or heteromeric multimeric E3s [9].

## 3. The Role of the UPS in Plant Stress Response

The UPS is a key regulator of plant proteome homeostasis. In response to environmental stress, the UPS can regulate gene expression and cellular responses via ubiquitination of regulatory proteins such as transcription factors involved in environmental stress responses [23]. Many genes encoding components involved in the UPS are induced by abiotic stress [3].

The role of various E3s in regulating stress signaling is largely dependent on the function of its substrate and is influenced by its ability to bind to the substrate proteins [9]. For example, interaction of an E3 and its substrate may only occur under normal conditions or conversely only during abiotic stresses, leading to different physiological outcomes. Dehydration-responsive element-binding protein 2A (DREB2A) is a key transcription factor that regulates the expression of numerous genes which are responsive to heat, drought, and salt stresses in plants [24,25,26]. Under non-stress conditions, DREB2A is ubiquitinated by RING E3s, DRIP1 (DREB2A-INTERACTING PROTEIN1), and DRIP2, and subsequently degraded [25]. Whereas under stress conditions, an A20/AN1-type zinc finger protein, AtSAP5 (Stress Associated Protein 5), functions as a ubiquitin ligase that mediates the ubiquitination and degradation of DRIP1/2 to release and stabilize DREB2A and subsequently induce the expression of downstream responsive genes [27]. Additionally, INDUCER OF CBF EXPRESSION1 (ICE1) can positively regulate the expression of *C-REPEAT BINDING FACTOR/DRE BINDING FACTOR1* (*CBF*/*DREB1*), which encodes a transcription factor that activates a series of Cold-Regulated (*COR*) genes in response to cold stress [28,29]. The stability of ICE1 is crucial for its function in cold stress and is regulated through the UPS. In *Arabidopsis*, a RING protein HIGH EXPRESSION OF OSMOTICALLY RESPONSIVE GENES1 (HOS1) acts as an E3 ligase for the ubiquitination of ICE1, thus negatively regulating cold response. The interaction between ICE1 and HOS1 is blocked by the phosphorylation of ICE1, which is catalyzed by a member of the SNF1-related protein kinase family, OPEN STOMATA1 (OST1), thus, enhancing the stability of ICE1 and inducing cold tolerance [30].

**Figure 1:**
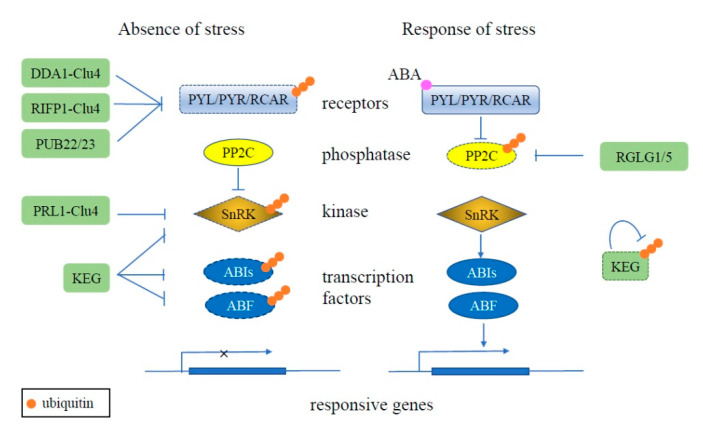
Schematic of ubiquitination regulating the plant ABA signaling pathway. Under normal conditions (left), the redundant ABA receptors PYL, PYR, and RCAR are ubiquitinated (orange circles) and degraded (dashed outline) by E3 ligases, such as DDA1-Clu4, RIFP1-Clu4, and PUB22/23. Kinase SnRK and transcription factors ABI5 and ABF also are degraded via the UPS, mediated by RING E3 KEG. Together, these result in the ABA pathway being inactivated in the absence of stress. During stress conditions (right), the ABA receptors are activated by the binding of ABA, whereby they then interact with PP2C to inhibit its activity. PP2C activity is also inhibited by the RING E3s RGLG1 and RGLG5 and degraded via the UPS. KEG also undergoes proteasome-dependent degradation dependent on its own ligase activity, resulting in stabilization of SnRK, ABI5, and ABF and activation of the ABA signaling pathway throughinduction of responsive gene expression.

The UPS can also play important roles is regulating hormone pathways in plants. The ABA (abscisic acid) signal pathway includes multiple receptors PYR1 (pyrabactin resistance 1)/PYL (PYR1-like)/RCAR (regulatory components of ABA receptors), protein phosphatase type 2Cs (PP2Cs), SnRKs (Sucrose non-fermenting related kinase), and transcription factors ABI5 (ABA INSENSITIVE 5) and ABF (ABRE-binding factor). The PP2Cs, SnRKs, and transcription factors are activated successively by the binding of ABA to the receptors under abiotic stress conditions, including drought, salt, and cold stresses [31]. Under normal conditions, the UPS plays a crucial role in inhibiting the ABA signal pathway by degrading some of its components. Deetiolated 1 (DET1) and DDB1-associated protein 1 (DDA1) and a F-box protein RIFP1 (RCAR3 INTERACTING F-BOX PROTEIN 1) act as the substrate receptor and recruiting component for the RING E3 Clu4 to mediate the ubiquitination and degradation of PYL8, PYL4, and PYL9 [32,33]. PYL9 can also interact with PUB (Plant U-Box) E3s, PUB22 and PUB23, and is ubiquitinated for degradation [34]. The members of the SnRK superfamily, SnRK1, SnRK2 and SnRK3, play crucial roles in activating ABA responsive transcription factors. Under normal conditions, in addition to being inactivated by phosphatase PP2C, SnRKs can interact with pleiotropic regulatory locus 1 (PRL1), which may function as the substrate-binding component of a Cul4 (CULLIN 4)-based RING E3, to be ubiquitinated for degradation [35]. The ubiquitination and proteasomal degradation of SnRK3.26 can also be promoted by interaction with RING E3 Keep on Going (KEG) [36]. Some transcription factors involved in ABA signaling pathway, such as ABI5, ABF1, and ABF3, can be ubiquitinated via a RING E3, KEG to be proteasomally degraded [37,38,39]. The ABA signaling pathway is activated by the binding of ABA to the PYR1/PLY/RCAR receptors, which interact with and inhibit the phosphatase PP2C [40]. PP2C activity is also inhibited by the RING E3s RGLG1 (RING DOMAIN LIGASE 1) and RGLG5 via the UPS [41]. Meanwhile, the proteasome-dependent degradation of KEG requires its own ubiquitin ligase activity and subsequently leads to the accumulation of SnRK3.26. The self-ubiquitination of KEG is phosphorylation-dependent, which is catalyzed by CBL-Interacting Protein Kinase 26 [42]. Thus, in plants, the UPS is involved throughout the ABA signaling pathway to ensure the correct timing of the response (Figure 1).

**Figure 2:**
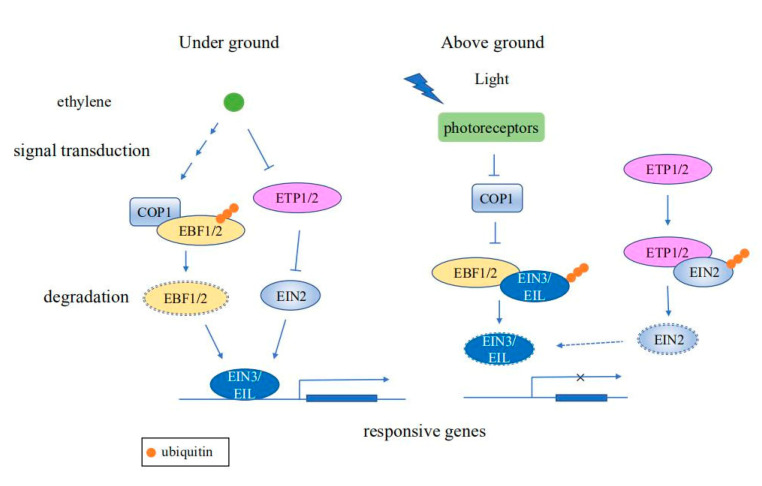
Schematic diagram of ubiquitination regulating ethylene signaling pathways. When seedlings grow underground, ethylene signaling promotes the ubiquitination of EBF1/2 by the E3 ligase COP1. In the absence of EBF1/2, EIN3/EIL is stabilized, resulting in induction of expression of ethylene responsive genes. Ethylene reduces the protein levels of ETP1/2, thus stabilizing EIN2, which in turn promotes the expression of ethylene responsive genes. As seedlings grow toward the soil surface, regulation by photoreceptors causes COP1 activity to decrease gradually, resulting in ubiquitination and subsequent degradation of EIN3/EIL by EBF1/2, causing termination of the ethylene response. Moreover, EIN2 is ubiquitinated and degraded by ETP1/2 to repress the ethylene response.

Ethylene signaling is important for regulating abiotic stress responses and numerous plant developmental processes, including germination, soil emergence, fruit ripening, and senescence [43]. The expression of ethylene signaling responsive genes are largely regulated by the transcription factors ETHYLENE INSENSITIVE3 (EIN3) and EIN3-Like (EIL) [44]. The stability of EIN3 and EIL1 are regulated by the coaction of ubiquitin-ligases EIN3 BINDING F-BOX1 (EBF1) and EBF2 [45]. After seed germination, EIN3/EIL1 are stabilized by both light and ethylene signaling to promote the underground growth of seedlings. An E3 ubiquitin ligase CONSTITUTIVE PHOTOMORPHOGENIC 1 (COP1) functions as a central repressor of light signaling to mediate the ubiquitination and degradation of EBF1/2 [46]. As seedlings grow toward the soil surface, COP1 activity, which is negatively regulated by photoreceptors, gradually decreases, resulting in attenuation of the ethylene response [47]. Moreover, another key regulator of the ethylene signaling pathway, ETHYLENE-INSENSITIVE2 (EIN2), can also be regulated via ubiquitination and degradation. The ethylene response can be repressed by ubiquitination of the integral membrane protein EIN2 by EIN2 TARGETING PROTEIN1 (ETP1) and ETP2. However, ETP abundance is negatively regulated by ethylene, contributing to activation of the ethylene response in the presence of ethylene [48] (Figure 2).

In addition, the plant auxin signaling pathway is also regulated by the UPS. In the presence of auxin, the transcriptional repressors AUXIN/INDOLE-3-ACETIC ACID (AUX/IAA) are targeted for degradation by SKP1-CULLIN1-F-BOX (SCF) ubiquitin-protein ligases to turn on the auxin signaling pathway [49]. However, further study is needed to uncover the intricacies of the relationship between ubiquitination and auxin signaling.

## 4. Ubiquitin-Mediated Regulation of Autophagy in Plants

Found in all eukaryotic cells, autophagy is a pathway to degrade cytoplasmic content. This is achieved by forming specialized autophagic vesicles which are delivered to the vacuole for degradation [50]. There are three types of autophagy which have been described in plants: microautophagy, macroautophagy, and mega-autophagy [51]. During microautophagy, cellular contents congregate at the vacuole surface and are trapped by invagination of the vacuole for degradation. Mega-autophagy is an extreme form of autophagy in which the vacuole ruptures to release hydrolases into the cytoplasm, ultimately resulting in programmed cell death [8,51]. The mechanism of macroautophagy, or autophagy for short hereafter, is well characterized in yeast and occurs in several distinct steps, requiring a series of ATGs [8]: (1) The dephosphorylation of ATG13 caused by the inactivation of TOR (Target of Rapamycin) and the hyperphosphorylation of ATG1 catalyzed by other kinases lead to the formation of the ATG1-ATG13-ATG11-ATG101 complex to initiate autophagy; (2) Activated ATG1 kinase promotes ATG9-mediated delivery of lipids to the developing phagophore; (3) ATG8s, a marker of autophagy, are attached to the autophagosome membrane by the ATG5-ATG12-ATG16 E3 ligase complex via the ubiquitin-like pathway; and (4) the autophagosome is delivered to the vacuole for degradation. The orthologs of most yeast ATG proteins have been identified in plants, indicating that the mechanism of autophagy may be well conserved in plants and yeast.

**Figure 3:**
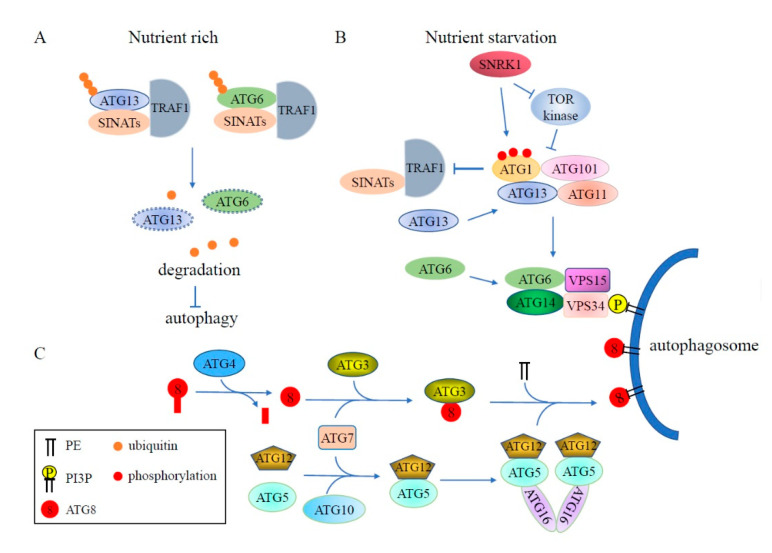
Schematic diagram of ubiquitination involved in the regulation of plant autophagy. (**A**) Under nutrient rich conditions, ATG13 and ATG6 are ubiquitinated by RING-type E3 ligases SINATs with the assistance of TRAF1 and then degraded via the 26S proteasome, leading to inhibition of autophagy. (**B**) During nutrient starvation, TRAF1 stability is impaired by activated ATG1, resulting in accumulation of ATG13 and ATG6 and formation of autophagosomes. (**C**) ATG8 is labeled into the autophagosome membrane in a similar way as the ubiquitination pathway. The ubiquitin-like protein ATG8 is cleaved by Cys protease ATG4 to expose a conserved C-terminal glycine, then ATG8 is transferred to the E2-like enzyme ATG3 through E1-like ATG7, and ultimately, labeled into the autophagosome membrane as ATG8-PE by E3 ligase complex ATG5-ATG12-ATG16. The decoration of the phagophore with PI3P is catalyzed by a complex containing ATG6/ATG14/VPS15/VPS34. PE: phosphatidylethanolamine; PI3P: phosphatidylinositol-3-phosphate.

The UPS and autophagy, the two major cellular degradative pathways controlling the stability of the cellular proteome, employ separate molecular machinery. The UPS specifically degrades substrates via the E1-E2-E3 system which conjugates ubiquitin on to the substrates, whereas autophagy is a vesicular trafficking pathway that transfers damaged or redundant proteins and organelles to the vacuole or lysosome [52,53,54]. Because of this, the UPS and autophagy pathways have long been thought of as parallel processes with no intersection. However, it now appears that autophagy and the UPS are interconnected and may mutually affect each other (Figure 3).

### 4.1. Regulation of Autophagy by Ubiquitination

In plants, the assembly of the ATG1-ATG13 complex promotes autophagy initiation. The activity of the ATG1-ATG13 complex is negatively regulated by TOR kinase in response to nutrient conditions. Under starvation conditions, the levels of phosphorylated ATG1a and ATG13a drop dramatically, but this turnover is abolished when the ATG system is inhibited, indicating that the ATG1-ATG13 complex is degraded by autophagy [55]. Recent studies revealed that the stability of the ATG1-ATG13 complex is modulated by the ubiquitination pathway [56]. In *Arabidopsis* wild-type plants, ATG1a and ATG13a accumulate under carbon or nitrogen starvation, but are significantly reduced under prolonged starvation treatment. Degradation of ATG1a and ATG13a is repressed by treatment with the proteasome inhibitor MG132, indicating that the 26S proteasome modulates the stability of ATG1a and ATG13a. Further studies have shown that the ubiquitination of ATG13a is enhanced by overexpression of the RING-type E3 ligase SEVEN IN ABSENTIA OF ARABIDOPSIS THALIANA1 (SINAT1) in wild-type plants. However, this enhanced ubiquitination is impaired in the *traf1a/b* (tumor necrosis factor receptor-associated factor1a/b) mutant, demonstrating that TRAF1a and TRAF1b act as adaptors to mediate the ubiquitylation and degradation of ATG13a by SINAT1. Although ATG1 protein levels are also significantly reduced during prolonged starvation treatment, the mechanism of ATG1 degradation is still elusive. It was shown that the stability of TRAF1 can be regulated by ATG1, indicating feedback regulation between the ATG1-ATG13 kinase complex and TRAF1 proteins [56,57].

In addition, in *Arabidopsis*, TRAF and SINAT proteins are also involved in regulating ATG6 stability, which subsequently mediates autophagosome formation [57,58]. TRAF1a and TRAF1b can interact directly with ATG6. Loss of *TRAF1a* and *TRAF1b* causes stabilization of ATG6 protein levels by reducing its ubiquitination level. It was also found that SINAT1 and SINAT2 can interact with and ubiquitinate ATG6. In the *traf1a/b* mutant, SINAT1- and SINAT2-induced degradation of ATG6 is impaired, indicating that TRAF1a and TRAF1b function as adaptors required for SINAT1/SINAT2-mediated ubiquitination and degradation of ATG6 [58].

SINATs (SINAT1, SINAT2, SINAT3, and SINAT4) are primarily localized to the endosomal and autophagic vesicles and are involved in the ubiquitination and proteasomal degradation of FYVE DOMAIN PROTEIN REQUIRED FOR ENDOSOMAL SORTING 1 (FREE1) and VACUOLAR PROTEIN SORTING 23A (VPS23A). FREE1 and VPS23A are key components of the plant specific ESCRT (endosomal sorting complex required for transport), which is involved in endosomal sorting and autophagic degradation [59,60]. Furthermore, the SINATs can be co-degraded with FREE1 and VPS23A via the vacuolar pathway and expression of SINATs promotes increased sensitivity to ABA and induction of ABA-responsive gene expression. Together, these findings indicate that SINATs-mediated degradation of FREE1 and VPS23A may be involved in the regulation of ABA signaling [59]. In addition, the protein levels of SINAT1-4 are induced by iron deficiency, promoting the ubiquitination and degradation of FREE1 and subsequently relieving the repression of FREE1 on iron absorption [60]. These studies suggest that SINAT-mediated ubiquitination plays important roles in autophagy and participates in the regulation of important physiological processes in plants.

### 4.2. Ubiquitin-Like Systems in Autophagy

The ubiquitin-like conjugation system is comprised of E1-like ATG7, E2-like ATG3, and the E3-like ATG5-ATG12-ATG16 complex, and is conserved in all eukaryotes [8,61,62]. It is involved in the formation of autophagic vesicles via mediation of ATG8 lipidation. This process starts with the cleavage of the ubiquitin-like protein ATG8 by the Cys protease ATG4 to expose a conserved C-terminal glycine, which is essential for the subsequent conjugation reaction [61,63]. Subsequently, ATG8 is activated at this glycine by the ATP-dependent activating enzyme, E1-like ATG7, to form a thioester bond with the Cys residue of ATG7 [64]. Then, ATG8 is transferred by the E2-like enzyme ATG3 to its Cys residue [62]. Finally, ATG8 is covalently conjugated to the lipid molecule phosphatidylethanolamine (PE) and attached to the phagophore membrane, mediated by the E3 ligase complex ATG5-ATG12-ATG16, via an amide bond between the C-terminal Gly residue of ATG8 and the amino group of PE [8,65].

To form the ATG5-ATG12-ATG16 E3 ligase complex, the Ub-fold protein ATG12 is first adenylated at its C-terminal glycine by E1-like activating enzyme ATG7 to bind via a thioester bond to the active-site Cys in ATG7 [64,66]. Then, the activated ATG12 is transferred to the active-site Cys of E2 conjugating enzyme ATG10 by transesterification and subsequently conjugated to a conserved lysine residue of ATG5 via a peptide bond [67]. The ATG12-ATG5 conjugate then assembles with the dimeric scaffold protein ATG16 to function as an E3-like ligase in ATG8 lipidation [68,69]. ATG8s decorate the autophagosome membrane and serve as an anchor for autophagy receptors bringing cargo to the autophagosome, similar to how the UPS labels substrates to be degraded.

### 4.3. Ubiquitination in Selective Autophagy

**Figure 4:**
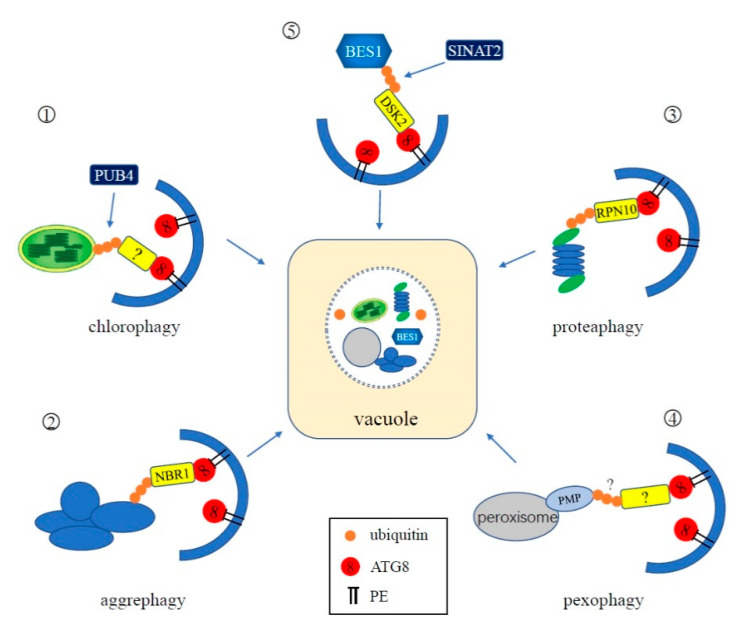
Schematic representation of the interplay between ubiquitination and selective autophagy. ①: In chlorophagy, the surface of an aberrant chloroplast is polyubiquitinated by cytoplasmic-localized E3 ligase Plant U-Box 4 (PUB4). An unknown adapter mediates the binding of ubiquitinated chloroplasts and ATG8 for degradation. ②: NBR1 is the adaptor of aggrephagy in plants. It can simultaneously interact with ubiquitin attached to the aggregates and ATG8. ③: The proteasome itself can be ubiquitinated and degraded by autophagy, during which RPN10 functions as a ubiquitin receptor of proteaphagy in plants. ④: In plants, an unknown pexophagy receptor likely interacts with ubiquitinated peroxisomal membrane proteins, mediating the degradation of peroxisomes. ⑤: In *Arabidopsis*, BES1 is ubiquitinated by E3 ligase SINAT2 and is subsequently recognized by selective autophagy receptor protein DSK2 for degradation through autophagy.

Many studies have demonstrated that cellular substrates and organelles—including chloroplasts, peroxisomes, mitochondria, the proteasome, nucleus, protein aggregates, and pathogens—can be selectively degraded via autophagy [8]. Some selective autophagy receptors have been found to be ubiquitinated during the course of degrading their respective selective substrates or organelles [8], indicating that the UPS plays an important role in selective autophagy (Figure 4).

#### 4.3.1. Chlorophagy

Chloroplasts are specialized plastid organelles found exclusively in plants and play an important role in photosynthesis and other metabolic functions necessary for plant growth and development [70]. Damaged chloroplasts are degraded via selective autophagy, termed chlorophagy, for quality control and remobilization of nitrogen and fixed carbon [71,72]. Depending on the status of the chloroplasts and the nutritional needs of the plant, chlorophagy can occur through several different mechanisms, including packaging of whole chloroplasts, budding of stromal proteins into autophagic vesicles called Rubisco-containing bodies (RCBs), or via ATI1 (ATG8-INTERACTING PROTEIN1)-decorated plastid bodies [8].

Ubiquitination is involved in the degradation of whole chloroplasts, in which entire chloroplasts are encapsulated into ATG8-decorated autophagic vesicles [71]. This process is promoted by strong photo-oxidative damage of chloroplasts caused by UV-B and high-intensity visible light [71]. Recent studies reveal that photo-damaged chloroplasts are associated with excess reactive oxygen species (ROS). Damaged proteins on the envelope membranes of aberrant chloroplasts are polyubiquitinated and the ubiquitylated chloroplasts are recognized by unknown autophagic receptors to form autophagy vesicles and delivered to the vacuole for degradation [73]. It has been reported that a cytoplasmic-localized E3 ligase PUB4 (Plant U-Box 4) is involved in the ubiquitination of chloroplast membrane proteins, but the specific targets of PUB4 have not been identified, nor is it clear if they are damaged [73]. Interestingly, PUB4 is not necessary for the induction of chlorophagy since chlorophagy can be induced by high light and low temperature treatment in the *pub4* mutant [74]. However, the double *atg5 pub4* mutant results in accelerated leaf chlorosis during senescence and reduced seed production, suggesting that both autophagy and the ubiquitination activity of PUB4 contribute to protein degradation in senescing leaves. Thus, PUB4 and autophagy may function in two semi-redundant pathways to remove chloroplasts [74]. The TOC (translocon at the outer envelope of chloroplasts) complexes in the plastid outer membrane have been shown to be ubiquitinated by a RING-type ubiquitin E3 ligase, SP1 (suppressor of ppi1 locus1), during chloroplast development [75]. However, recent evidence suggest that SP1-mediated ubiquitination may be not involved in chlorophagy [75].

#### 4.3.2. Aggrephagy

Cellular proteins must fold into their correct conformation to function properly. However, under various stresses, these proteins can become misfolded or damaged and become toxic to cell. The UPS is considered the primary degradation process to remove ubiquitinated misfolded and damaged proteins, but recent studies have uncovered that a form of selective autophagy called aggrephagy plays an important role in degrading ubiquitinated protein aggregates for quality control [8].

In mammals, the autophagy receptors p62 and NBR1 (NEIGHBOR OF BRCA 1) can bind ubiquitinated aggregates via their ubiquitin binding domains (UBA) and tether microtubule-associated protein 1 light chain 3 (LC3), the mammalian ATG8 homolog, through their LC3-interacting region (LIR) motifs to facilitate selective sequestration of ubiquitin condensates to the autophagosome for clearance [76,77]. Loss of function of *Arabidopsis* NBR1, a homolog of mammalian p62 and NBR1, causes increased sensitivity to abiotic stresses and accumulation of insoluble protein aggregates. *Arabidopsis* NBR1 binds ubiquitin through a C-terminal UBA-domain and interacts with *Arabidopsis* ATG8, via the conserved LIR motif, indicating that NBR1 functions as the selective autophagy cargo adaptor of the aggrephagy pathway in plants [78,79].

In *Arabidopsis*, a chaperone-associated E3 ubiquitin ligase, CHIP (carboxyl terminus of Hsc70-interacting protein), which mediates degradation of nonnative proteins by 26S proteasomes, participates in aggrephagy during stress response. While *chip* mutants are similarly sensitive to abiotic stresses as *nbr1* mutants, *chip nbr1* double mutants display increased sensitivity, suggesting cumulative roles for CHIP and NBR1 [80]. However, protein aggregates induced by stress are still ubiquitinated in *chip* mutants. After a relatively short period of heat stress, different types of protein aggregates accumulate in *nbr1* and *chip* single mutants [80]. For example, rubisco activase and catalases are preferentially accumulated in the *nbr1* mutant while light-harvesting complex proteins accumulate at high levels in the *chip* mutant. These findings indicate that CHIP and NBR1 mediate two distinct but complementary anti-proteotoxic pathways under stress conditions [80]. Additionally, NBR1 can also interact with the ABA signaling regulatory proteins ABI3, ABI4, and ABI5, indicating that the ubiquitinated ABIs may be degraded by autophagy [81].

#### 4.3.3. Proteaphagy

In eukaryotes, the proteasome contains a core particle (CP) and a regulatory particle (RP) [82]. The 20S CP consists of four heteroheptameric rings and is responsible for the entrance of substrates and removal of degradation products. The 19S RP is a multifunctional complex that functions as the proteasome cap and mediates substrate recognition, binding, unfolding, and translocation to the CP [82]. Despite the ability to degrade ubiquitylated proteins via the UPS, the proteasome itself can be degraded by autophagy, termed as proteaphagy.

Proteaphagy was first discovered in *Arabidopsis*, but is conserved in yeast and mammals [83,84,85]. In *Arabidopsis atg* mutants, such as *atg1*, *atg4*, *atg5*, *atg7*, *atg12*, and *atg13*, the protein levels of the 26S proteasome subunits are significantly increased [85]. Fluorescently labeled CP subunit PAG1 (proteasome α subunit G1) and the RP subunit RPN5 were found to colocalize with autophagic vesicles and eventually appear in the vacuole under nitrogen starvation. Moreover, ubiquitination of the proteasome has been detected and the deposition of proteasomes into the vacuole is induced when plants are treated with the proteasome inhibitor MG132, indicating that whole proteasomes are ubiquitylated and eliminated through autophagy [85]. The ubiquitin receptors of proteaphagy, Cue5, p62, and RPN10, have been identified in yeast, mammals, and plants, respectively [83,84,85]. RPN10 contains two UIM (ubiquitin-interacting motif) domains at its C-terminus, allowing RPN10 to simultaneously bind to ubiquitylated proteasomes through one UIM and ATG8 via another [85]. In *Arabidopsis* mutants lacking the C-terminal domain of RPN10, MG132-induced selective proteaphagy is blocked, demonstrating that RPN10 functions as a ubiquitin receptor of proteaphagy [85].

#### 4.3.4. Pexophagy

Peroxisomes universally exist in all eukaryotes and function in fatty acid β-oxidation, glyoxylate cycles, photorespiration, and other metabolic processes which are involved in plant growth, development, and response to various stresses [86,87,88]. Damaged, obsolete, or redundant peroxisomes need to be eliminated by a selective autophagy pathway termed pexophagy to maintain cellular redox homeostasis [89,90]. In yeast, the selective receptors of pexophagy are Atg30 (*Pichia pastoris*) and Atg36 *(Saccharomyces cerevisiae)*, which recognize peroxisomes to be degraded by direct interaction with peroxisomal membrane proteins, such as Pex3 (peroxin 3) and Pex14 [91,92]. However, no evidence has demonstrated that ubiquitination is required for yeast pexophagy. Interestingly, in mammals, the pexophagy receptors NBR1 and p62 have the ability to recognize ubiquitylated peroxisomal proteins, suggesting that ubiquitination is involved in mammalian pexophagy [93]. In mammals, overexpression of PEX3 or PEX2 induces peroxisome ubiquitination and degradation through NBR1-mediated pexophagy [94,95]. These findings demonstrate that ubiquitination of peroxisomal membrane proteins can trigger pexophagy in mammals. Moreover, recent evidence suggests that ubiquitinated mammalian Pex5 serves as a potential peroxisome degradation signal, which is recognized by autophagy receptor, p62 [96]. Similar to yeast pexophagy, the role of ubiquitination has not yet been established in plant pexophagy. Nevertheless, in *Arabidopsis,* the ubiquitin receptor protein DOMINANT SUPPRESSOR OF KAR2 (DSK2) was found to interact with PEX2 and PEX12 and has been suggested to participate in the peroxisomal membrane-associated protein degradation pathway [97]. As a selective autophagy receptor, DSK2 recognizes and targets ubiquitinated BRI1-EMS SUPPRESSOR1 (BES1) for autophagy in response to drought and starvation stresses (see Section 4.3.5). However, whether DSK2 serves as a potential pexophagy receptor and whether ubiquitination is involved in plant pexophagy are still not clear and need further investigation.

#### 4.3.5. Others

In *Arabidopsis*, ubiquitinated BES1, which is a key transcription factor regulating gene expression in the brassinosteroid signal pathway, accumulates under treatment with proteasome and autophagy inhibitors, indicating that BES1 can be degraded through the UPS and autophagy [98,99]. Further exploration revealed that ubiquitin-binding and selective autophagy receptor protein DSK2 can interact with both BES1 and ATG8 to mediate BES1 degradation through autophagy [99]. In addition, the E3 ligase SINAT2 can interact with DSK2 to form a complex to target ubiquitinated BES1 for the UPS under starvation conditions [99]. In summary, ubiquitin modifications can regulate plant selective autophagy. Hence, the UPS and autophagy cooperate and complement each other during plant development and stress response to jointly regulate cellular homeostasis.

## 5. Future Perspectives

As complex and well-regulated pathways, the UPS and autophagy act together to form an integrated quality control network in response to general cellular stress. Although their mechanisms vary widely, there is growing evidence that they are closely related. Ubiquitin, as a molecular marker, not only plays a role in the UPS pathway, but also mediates the initiation of many autophagic processes. Furthermore, the components of the UPS can be degraded by autophagy and vice versa. While this area of study has been well characterized in mammals, it is just beginning to be revealed in plants, leaving many mysteries to be solved. For example, under stress, the UPS and autophagy pathways may both need to be activated to withstand adverse environments. Thus, how do plants coordinate these different, interconnected pathways? What determines whether ubiquitinated protein substrates are degraded by UPS or by autophagy? As proteolysis plays a central role in various biological processes of plants, our understanding on the relationship between the UPS and autophagy will aid in the understanding of the molecular mechanisms of plant development and stress response.
